# Facilitators and Barriers for a Good Night’s Sleep Among Adolescents

**DOI:** 10.3389/fnins.2020.00092

**Published:** 2020-02-07

**Authors:** Gita Hedin, Annika Norell-Clarke, Peter Hagell, Hanne Tønnesen, Albert Westergren, Pernilla Garmy

**Affiliations:** ^1^Centre for Research on Child and Adolescent Mental Health, Karlstad University, Karlstad, Sweden; ^2^Faculty of Medicine, Lund University, Lund, Sweden; ^3^Faculty of Health and Science, Kristianstad University, Kristianstad, Sweden

**Keywords:** adolescents, electronic media use, focus group interviews, health, sleep, qualitative content analysis

## Abstract

**Background:**

Sleep deprivation among adolescents is a major public health issue. Although previous studies have described their sleep habits and the consequences thereof, the voices of adolescents themselves are rarely heard. The aim of this study was to investigate adolescents’ experiences regarding what they perceived as facilitators and barriers for a good night’s sleep.

**Methods:**

A qualitative focus group study with Swedish adolescents (*n* = 45) aged 16–18 years was performed with seven focus groups and analyzed using qualitative content analysis.

**Results:**

Three categories were identified in the analysis regarding facilitators and barriers for achieving a good night’s sleep: (1) Striving for a sense of well-being, (2) Tiring yourself out, and (3) Regulating electronic media availability. The adolescents thought that sleep was important in order to be able to cope with everyday life and to allow physical recovery. Overall, the adolescents were knowledgeable regarding commonly recommended strategies for improving sleep, but they had trouble finding a balance between sleep and other activities. Electronic media was used to obtain a sense of belonging and to communicate with others, which in itself was described as important for the adolescents’ well-being. However, communicating with friends and family during the night conflicted with achieving a good night’s sleep. Parental behaviors (late work habits, internet rules) were also perceived as important for adolescents’ sleep habits.

**Conclusions:**

An understanding of the dilemma of finding a balance between sleep and other activities may aid future sleep-promoting interventions for adolescents, incorporating the impact from social factors’ on the adolescents’ sleep.

## Facilitators and Barriers for a Good Night’s Sleep Among Adolescents

Population-based studies indicate that approximately 25–35% of adolescents get insufficient sleep, and these estimates are increasing (Gradisar et al., 2011). The sleep recommendations for adolescents are 8–10 h, and below this range, lack of sleep may affect health and well-being (Hirshkowitz et al., 2015). It has been found that 61% of adolescents in North America are tired during school. Sleep length among adolescents from North America, Europe and Asia, range from 7.4–8.4 h (Gradisar et al., 2011). In an interview study with 14-year-old adolescents, Gruber et al. (2017) highlighted facilitators for sleep as physical, as well as relaxing activities before sleep. Barriers to sleep were stress, anxiety, and the use of electronic media before sleep. Adolescents were aware of the negative consequences of poor sleep (Gruber et al., 2017). During recent decades, sleep patterns have changed among Swedish adolescents toward later bedtimes (Norell-Clarke and Hagquist, 2017), yet few studies have investigated the opinions of adolescents regarding what affects their sleep. This study therefore aims to better understand how adolescents perceive facilitators and barriers for a good night’s sleep.

Adequate sleep duration is associated with better attention, behavior, cognitive functioning, emotional regulation, and physical health among children and adolescents (Paruthi et al., 2016). Conversely, insufficient sleep has been associated with health problems as well as sleepiness in class, concentration difficulties, difficulties in school and poorer grades (Paruthi et al., 2016). Electronic media use has been found to be one reason for insufficient sleep (Cain and Gradisar, 2010). Adolescents who had inadequate sleep across the school week also reported problematic levels of sleepiness, fatigue, depressed mood, and anxiety (Short et al., 2013). Limited sleep and lack of sleep during adolescence can affect sleep later in life (Robotham, 2011). Adolescence and its developmental periods are characterized by many changes, and some problems during these developmental periods include problematic sleep habits (Wolfson and Carskadon, 2003). Besides this, Wolfson and Carskadon (2003) concluded that adolescents who have insufficient sleep are more likely to use stimulants like caffeine and nicotine to get through the day. Also, late bedtimes, as defined as 12:00 AM and later on a weekday, may be a risk factor for the prevalence of diabetes mellitus (Yan et al., 2019).

One way to change problematic sleep habits may be to gain a better understanding of the target population’s perception of sleep and what promotes and hinders a good night’s sleep. In this perspective, a salutogenic approach that emphasizes health promoting resources (Bauer et al., 2006) by inquiring about sleep promoting factors appears appropriate (Antonovsky and Sagy, 2017). The aim of this study was to investigate Swedish adolescents’ experiences regarding what they perceived as facilitators and barriers for a good night’s sleep.

## Materials and Methods

A qualitative focus group design was chosen to capture the experiences of adolescents.

Ethical approval for the study was obtained from the Regional Ethical Review Board in Lund, Sweden (EPN 2017/600). Focus group interviews were conducted to capture the various experiences of the adolescents as they provide the opportunity for a group of persons to describe and share their experiences on the selected topics. Focus group interviews are useful when trying to understand how a target group perceives and reasons regarding a phenomenon, because the format promotes reflection and differing opinions (Krueger and Casey, 2009).

### Sample

The study was conducted in seven upper secondary schools in rural and urban areas of southern and middle Sweden, and included public and private tuition free schools offering vocational as well as college preparatory tracks. In total, nine schools were contacted, but two of them declined participation due to lack of time. The participants (*n* = 45) comprised 28 girls and 17 boys in upper secondary school. The age of the adolescents were 16 years (*n* = 42) and 18 years (*n* = 3). The participants lived with their families (*n* = 38) or by themselves (in boarding schools or own apartments) during weekdays and with their families during weekends (*n* = 7).

### Data Collection

The schools distributed written information about the study to the students and their guardians. Thereafter, the teachers informed the students about the study orally and noted those who volunteered to participate, who then received written information about the study and its voluntary nature. All focus group interviews (*n* = 7 groups) were conducted during school days, between October 2018 and May 2019. About 3–8 people from each class participated in the study, so there were about 15 in each class who declined participation. The adolescents in each group were somewhat acquainted with one another since they were enrolled from the same classes. Both girls and boys participated in mixed groups that were led by the first, second, and/or last authors. The last author participated as an observer during the initial three focus group interviews. In order to increase the credibility of the study, the moderator fostered an open environment among the adolescents to ensure that all of the participants who wanted to talk were allowed to speak.

The time and place for the interviews was determined in consultation with school administrations and the students. The authors of this study developed a semi-structured interview guide (Appendix 1). Ten external experts (researchers) in the field of adolescent health, reviewed and gave feedback on the interview guide to optimize its purposefulness (Krueger and Casey, 2009). Following minor revisions, the interview guide was pilot tested in the first focus group interview. This did not lead to any further modifications, and the pilot interview was included in the final data set. Key questions in the interview guide included: “Could you please give examples of a good night’s sleep?”, “What are the challenges for a good night’s sleep?” and “How do you handle these challenges?” (Appendix 1). The focus group interviews lasted between 70 and 90 min in six cases, and one focus group interview lasted 45 min. All interviews were audio-recorded and transcribed verbatim. In studies that use semi-structured interviews and are analyzed using qualitative content analysis, the sample size is often justified based on interviewing participants until “data saturation” is reached (Francis et al., 2010). The interviews varied in duration due to the same reason.

### Analysis

The transcribed texts were analyzed using qualitative content analysis (Graneheim and Lundman, 2004). This technique makes it possible to analyse relatively large amounts of data while also focusing on variations within the data. Qualitative content analysis is composed of descriptions of the concrete content and interpretations of the abstracted content while focusing on subjects’ experiences (Graneheim and Lundman, 2004). The Nvivo Plus software, version 12 was used to organize and sort the text.

First, the transcripts were read several times to obtain a general sense of the information. The transcripts were then condensed and coded. Next, the codes were grouped into subcategories, which, in turn, were abstracted into categories. Then, categories and subcategories were discussed among the authors until consensus was reached. To strengthen the consistency of the analysis process across all of the transcripts, five members of the research team extracted a random sample of the data to evaluate the analysis at regular intervals during the analysis process. This was done in accordance with Guba (1981), since establishing credibility enhances the trustworthiness of the study.

## Results

**FIGURE 1:**
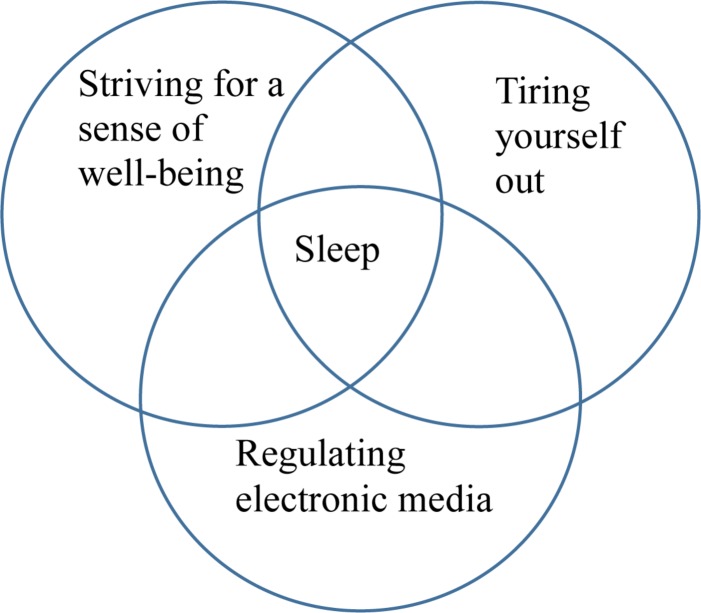
The three categories that interactively affect nighttime sleep according to Swedish adolescents.

Overall, the adolescents thought that sleep was important in order to be able to cope with everyday life and to allow physical recovery. Three categories emerged, which describe the adolescents’ experiences of facilitators and barriers for a good night’s sleep: (1) Striving for a sense of well-being, (2) Tiring yourself out, and (3) Regulating electronic media availability (Table 1). The three categories interactively affect nighttime sleep according to (Figure 1).

**TABLE 1:** Categories, subcategories, and codes based on focus group interviews with adolescents regarding what they perceive as facilitators and barriers for a good night’s sleep.

Category	Sub-category	Codes
Striving for a sense of well-being	Engagement in relaxing activities	Bodily self-care, mentally self-care, supporting friends
	Dealing with strains	Pain, worries, thoughts about life, conflicts with peers and family, stress in school, solving problems
Tiring yourself out	Exhausting oneself physically	Being engaged in sports, daily activities, and physical activities in school
	Being mentally wound up but sedentary	Being trapped with electronic media, schoolwork, leisure time
Regulating electronic media availability	Sense of relief	Sense of control, parental responsibility, technical solutions
	Feeling of losing control	Stress, difficult to find balance, letting other people down

### Striving for Sense of Well-Being

The adolescents described a striving for a sense of well-being linked with a good night’s sleep. This meant engaging in relaxing activities and dealing with strains.

*The engagement in relaxing activities* was described as beneficial for sleep. When they relaxed, they felt a sense of well-being. Relaxation meant different things to the adolescents, but some of them suggested that mental and bodily self-care was helpful. One common strategy to relax was to nap for a few hours after school. The adolescents stated that this napping routine was crucial after an exhausting day at school. However, they also described how this routine made it more difficult to relax later when it was time to fall asleep. Other reported relaxing activities included reading books or listening to audiobooks, podcasts, or music. A cold and dark bedroom was regarded as relaxing before sleep. Some preferred taking a warm bath and bodily self-care with massage or mediation. Others stated that they could go to sleep without any special routine.

“Just stop thinking and shut everything out and imagine that you’re sinking into the bed like into a cloud and relax. Then it is much easier to fall asleep.” (female)

Another aspect was that the adolescents felt relaxed while being with family and friends, experiencing togetherness, which was a facilitator for sleep. Lastly, some adolescents described that going outside to smoke was a strategy to promote relaxation. They mentioned that smoking was not good for their health, but said that it felt good and relaxing in the moment. Also hanging out with friends while they smoked – even if they did not smoke themselves – was relaxing.

*Dealing with strains* could mean school stress, worries, and pain. The adolescents described that during school terms, they always had a lot of homework to finish. In addition, some of the adolescents participated in music programs, amateur theater or other leisure activities. Overall, stress, anxiety, and thoughts about school and friends and family all had negative impacts on sleep. Some of the adolescents experienced a great deal of stress and anxiety. The interviews demonstrated that both boys and girls considered negative thoughts, stress, and anxiety as barriers for a good night’s sleep.

Anxieties due to conflicts with family members and peers could originate on the Internet. Negative comments on social media could cause severe anxiety, especially if the adolescents did not know who the sender was. Those who were exposed to these comments and conflicts stated that their sleep was greatly affected due to difficulties to relax. The targets were very tired the next day and sometimes even felt anxious.

“If someone writes *mmm*. in a message, then I can stay awake for several hours at night and think about it. Is the person angry, annoyed or what does *mmm*. mean”? (female)

Adolescents also reported that pain and anxiety directly affected their sleep. For example, one of the adolescents had chronic pain.

“I’m in constant pain. So, how good I sleep or if I sleep at all, depends on how bad it is, and what I did during the day.” (male)

The adolescents also felt that some strains were outside their control—for example, they reported that taking care of younger siblings negatively affected their sleep, and they stated that friends who sent text messages or called at night also negatively affected their sleep. Parents were described as having an impact on sleep in different ways. Some parents worked a lot during the evenings and nights, and therefore it seemed natural for the adolescents to also stay up late to complete their homework. This led to compromised sleep and tiredness the next day. Another dimension was parental rules. Although the adolescents vividly explained that it could be rather annoying when their parents had opinions about their sleep habits, they also admitted that parental advice and discussions were supportive in creating healthy sleep habits.

“When my parents are nagging me to go to sleep, I get really annoyed, though I know that I need to sleep to not be exhausted the day after.” (male)

### Tiring Yourself Out

The adolescents described tiring yourself out as exhausting oneself physically and being mentally wound up.

*Exhausting oneself physically* was described as having a positive impact on sleep. The type of physical activity was of little significance. The frequency of engagement in physical activities varied from one to seven days a week. Some examples of exercise included going to the fitness center, riding horses, playing football, and walking or bicycling for transportation. While most exercise was undertaken outside their homes, some of the adolescents also mentioned participating in physical activity at home, such as running up and down the stairs or juggling. The adolescents stated that it was easier to settle down after physical activity, irrespectively of its intensity or how long it had lasted. The adolescents stated that they were simply exhausted when it was time to sleep they fell asleep without any effort, and it felt good.

“I sleep better, fall asleep more easily because I’ve used the body physically and it makes me feel good and tired.” (male)

The adolescents described that they felt an inner calm after physical activity, an absence of anxiety and that they slept better. The adolescents also reported that they liked physical activity during school. Some participated in physical activities very late in the evenings, and this led to late bedtimes. Therefore, they suggested more physical activity to be scheduled during school hours. The adolescents specifically reported that they needed physical activity or else they would easily become sedentary during the day and have difficulties falling asleep at night.

“You get motivated together with your friends to get physically active and you get tired in the evening, so you fall asleep more easily at night. Then you wake up completely rested and do not feel tired in school the next day.” (male)

*Being mentally wound up but sedentary* was something the adolescents experienced as barriers for a good night’s sleep. However, the most common barrier was that they became captivated in electronic media use and succumbed to sedentary behaviors. The effect of adolescents captivated in electronic media was that the adolescents felt tired during the day. They tried to solve the fatigue by sleeping after school, which meant that schoolwork and leisure time were postponed until later in the evening.

### Regulating Electronic Media Availability

The adolescents expressed that it is important to be inaccessible during the night as interruptions can prevent a good night’s sleep. They reported that they had different strategies to make themselves inaccessible during nighttime, and this helped them to feel in control.

To switch off the sound on their smartphones or leave the smartphone outside the bedroom, and instead engage in activities such as reading books could give a *sense of relief.*

“I go to bed early and I just lay there. No screen, I just lay there and try to sleep. I did not fall asleep immediately but after a few nights I fell asleep quite quickly without looking at the smartphone.” (female)

Other adolescents used their smartphones to listen to relaxing music and audiobooks, receive calls, watch shows, and participate on social media. They reported that watching comics and scrolling through social media for a short time before falling asleep was calming. The adolescents expressed different opinions on parental involvement. Some said that their parents had no idea about their activities on social media and how frequently they engaged with it during the night. They felt that they were in control, but at the same time, they mentioned that they were tired during the day and that they had difficulties putting their smartphone away. Some of the adolescents mentioned that they wished that their parents had more rules about the media use, because then it would be easier to put the smartphone away, and they would not have to make the decision for themselves. They felt that this would also help them to feel relief. One of the adolescents stated that his parents shut down their wireless internet connection at a certain time in the evening and that this had helped him to sleep better.

“It is good that they turn off the internet at a certain time, because then I will not have any chance to get stuck with the smartphone for too long at night.” (male)

However, the adolescents mentioned that it was difficult to find a balance between being uninterrupted and yet be available for their friends and family at night. The adolescents described a *feeling of losing control* as a feeling of responsibility to be available for friends and family. For example, adolescents who had a driving license felt a responsibility to be available during the night in case a friend needed to be picked up urgently. Particularly adolescents in boarding schools expressed that family members expected them to be accessible in case of urgent family issues or if they wanted to check on them.

“Imagine if I would switch off the smartphone at night and when I wake up in the morning and something horrible has happened that I missed just because I prioritized my sleep. No, it is not acceptable to do something like that.” (female)

Some of the adolescents expressed that their smartphone use was addictive, meaning that they were unable to stop looking at the display.

“The experience of having trouble sleeping can vary, but I find it difficult to fall asleep if I use social media before bedtime.” (female)

## Discussion

The aim of this study was to investigate what adolescents perceive as facilitators and barriers to a good night’s sleep. Historically, in the public discourse about adolescent sleep habits, there has been a moralistic attitude where adolescents are seen as hedonistic in their evening and night habits and unaware of what is best for them (Matricciani et al., 2012). Our findings suggest that today’s adolescents are aware of the importance of sleep for their well-being and that they have access to different strategies to achieve a good night’s sleep. However, they also described internal and external barriers that interfered with their sleep, some of which were on a relational level, which goes against the implicit assumption in sleep promotion that states that sleep is mostly affected by individual factors that one can control (Thorleifsdottir et al., 2002).

Besides Gruber et al.’s (2017) interview study with 18 adolescents (14 years old) in Canada, our study is one of few, to our knowledge, where adolescent voices have been directly heard regarding facilitators and barriers for sleep. The two studies are consistent regarding the importance of physical and relaxing activities for sleep, as well as the negative consequences of electronic media, stress, and anxiety before sleep”? A surprising finding was that altruistic relationship-related values had an influence on sleep habits, for example, in decisions to be electronically available in case of needing to help a friend or in case of urgent family news. The former may be understood from a developmental perspective as peer relationships become increasingly important during adolescence (Knoll et al., 2015). The latter was more surprising as the relationship to parents during adolescence is usually found to be characterized by a strive for autonomy. Similarly, it was also notable that some adolescents sought more rules from their parents regarding sleep habits.

If the parents imposed more boundaries (e.g., not allowing smartphones in some places or after certain times), the adolescents were unburdened of making that decision for themselves. This result indicates that parents might place too much responsibility on adolescents when it comes to electronic media use. The importance of parental influence was also demonstrated in a study that showed children and adolescents are more likely to follow parental regulations than advice from social media (Hiniker et al., 2016). This might not specific be to adolescents’ sleep behavior but indicates that parental behavior affects adolescents’ sleep. Another finding related to parental influence in the present study was that the adolescents described how they would stay up later if their parents also stayed up late. Taken together, our findings suggest that parental behavior is an important influence on adolescents’ sleep.

One barrier to a good night’s sleep was social media use. Our participants expressed mixed feelings about their use of social media. Some became anxious about missing out if they were not connected all of the time, and many described how they would be online for longer than they intended, which had a negative influence on sleep and the next day’s performance. Also, the adolescents found it difficult to find a balance between being sufficiently inaccessible for sleep and being available to stay connected with friends and family. Nowadays, a large part of social interactions take place over social media, and anyone who makes themselves inaccessible (for the purpose of relaxing or avoiding disturbances) risks missing important events or contact attempts to a point where prioritizing yourself by “turning off” was considered selfish. Even though social media engagement was perceived as beneficial for relationships in this study, there are studies that show its negative effects on health. Specifically, social media use has a negative impact on aggression, drug use, eating disorders, and school performance (Strasburger et al., 2010).

This brings us to another finding: the importance of being physically active during the day to be able to fall asleep. The adolescents said that their electronic media use led to sedentary behaviors and a mentally active mind, both which interfered with their quality of sleep. Various forms of physical exercise were mentioned as facilitators for sleep, which is in concordance with other studies that have demonstrated that exercise has beneficial effects on sleep duration [e.g., (Kredlow et al., 2015]. Another benefit of physical activity was that it allowed the adolescents to be together with their friends. Regrettably, exercise was difficult to prioritize after school hours, as homework was time consuming.

Despite their insights in what promotes and disturbs sleep, the adolescents also used some strategies that are counter-productive for sleep. They mentioned that smoking was a relaxation strategy and that they napped for a few hours directly after school: both which decrease sleepiness later in the night (Boehm et al., 2016).

### Strengths and Limitations

The focus group design was well suited for capturing the adolescent’s views and experiences regarding sleep. The place where the interviews took place could have affected the groups’ openness, even if the school and participants choose the place. In this case, all participants were interviewed within the schools. In order to increase the trustworthiness of the study, several of the authors participated in the analysis process, and we used the same questions in all focus groups. A clear strength of this study is its focus on adolescents’ own perceptions of facilitators and barriers to a good night’s sleep.

The study was conducted in schools in southern and middle Sweden, and the question of transferability remains. Background information about socio-economic status, sleep disturbance, or psychiatric history among adolescents is not available in this study. Therefore, we do not know how generalizable the findings are to a broader adolescent population. In qualitative content analysis according to Graneheim and Lundman (2004), the goal is not to obtain generalizability but to identify differences and similarities in the material. Study participation was voluntary and, therefore, it is difficult to know if the participating individuals differed from their peers in any particular way. It may be assumed that our results represent a broad variety of experiences. Further studies in other contexts are recommended.

In all but one focus group interview, the conversation progressed smoothly. In one interview, it was somewhat more difficult to achieve an active discussion climate, and this interview only lasted for 45 min. Individual interviews might have given more depth to the conversations. However, moderators were accustomed conversation leaders with experience working with children and young people and endeavored to foster a good conversation climate. Focus group interviews also have the advantage of capturing collective views in a dynamic environment that promotes reflection and differing opinions.

### Clinical Implications

A surprising finding was that altruistic relationship-related values were perceived to have an influence on adolescent sleep habits. The adolescents also expressed a wish to communicate and obtain support from parents and significant others. Parents and health professionals need to be aware and informed of the great responsibility that adolescents take for their friends. If friends and family members feel distressed or need help during the night, adolescents feel a responsibility to be available. Yet, this is unreasonably great burden for a teenager to bear, therefore, in sleep education programs, parents should also be included in the intervention. Gruber (2013) points out the importance of preventive sleep education programs for children and adolescents regarding, for example, relaxing behaviors.

Relatively small efforts have a positive effect on young people’s sleep habits (Bonnar et al., 2015). For example, limiting evening phone use among adolescents increased sleep duration by 21 min (Bartel et al., 2019). However, only a quarter of invited adolescents participated in the study by Bartel et al. (2019), which may indicate that many adolescents lack motivation to change their evening phone use. The adolescents in our study showed an interest and desire to discuss sleep and salutogenic strategies to cope with barriers to healthy sleep habits. The main findings suggest that adolescents are aware of the importance of sleep for their well-being and that they have access to different strategies to achieve a good night’s sleep. The focus group methodology in this study was appreciated by the adolescents, as they were afforded the opportunity to discuss a highly relevant topic with peers in a safe and non-judgmental way. Therefore, discussing such strategies and supporting adolescents in finding strategies to improve sleep habits is recommended.

## Conclusion

Our findings suggest that adolescents are aware of different strategies to facilitate sleep and that they perceive factors outside their control as barriers to their sleep, such as social demands, family habits, and difficulties prioritizing physical exercise. An implicit assumption in sleep promotion has been that sleep is mostly affected by individual factors. Thus, advice is given to the individual on how to change their habits. Our study highlights the relational aspects of sleep, in that the behaviors and expectations from friends and parents as well as parental rules are all perceived as influential for adolescents’ sleep habits. Continued research on the sleep patterns of adolescents and the associated facilitators and barriers is needed from a public health perspective. Ultimately, the results of this study may form a basis for future sleep interventions among adolescents, incorporating the social factors’ impact on the adolescents’ possibility to achieve a good night’s sleep.

## Data Availability Statement

The datasets generated for this study are available on request to the corresponding author.

## Ethics Statement

The studies involving human participants were reviewed and approved by the Etikprövningsnämnden, Lund. Written informed consent to participate in this study was provided by the participants’ legal guardian/next of kin.

## Author Contributions

GH, PG, PH, AN-C, and AW designed research and analyzed the data. GH, PG, and AN-C Performed the research. GH wrote the first draft of the manuscript. GH, PG, PH, AN-C, AW, and HT revised the manuscript and approved the final version.

## Conflict of Interest

The authors declare that the research was conducted in the absence of any commercial or financial relationships that could be construed as a potential conflict of interest.

## Appendix 1

### Interview Guide

Focus groups, adolescents in their first year of upper secondary school.

The aim of this study was to investigate Swedish adolescents’ experiences regarding what they perceived as facilitators and barriers for a good night’s sleep.

**Table:**

Key questions:	Could you please give examples of a good night’s sleep.
	What are the challenges for a good night’s sleep?
	How do you handle these challenges?
Closing questions and summary:	What resources do you think exist in your everyday life to meet your challenges regarding
	sleep?
	Is there something we have not asked about that you would like to add?
